# Supplementary material for: Dominant contribution of Asgard archaea to eukaryogenesis
Source: bioRxiv. 2025 Jul 14:2024.10.14.618318. Preprint. [Version 3] doi: 10.1101/2024.10.14.618318 (PMC12338589; doi:10.1101/2024.10.14.618318)
Supplement: Supplement 1 [file NIHPP2024.10.14.618318v3-supplement-1.pdf]

bioRxiv preprint doi: https://doi.org/10.1101/2024.10.14.618318; this version posted July 14, 2025. The copyright holder for this preprint (which was not certified by peer review) is the author/funder. This article is a US Government work. It is not subject to copyright under 17 USC 105 and is also made available for use under a CC0 license.

Extended Data

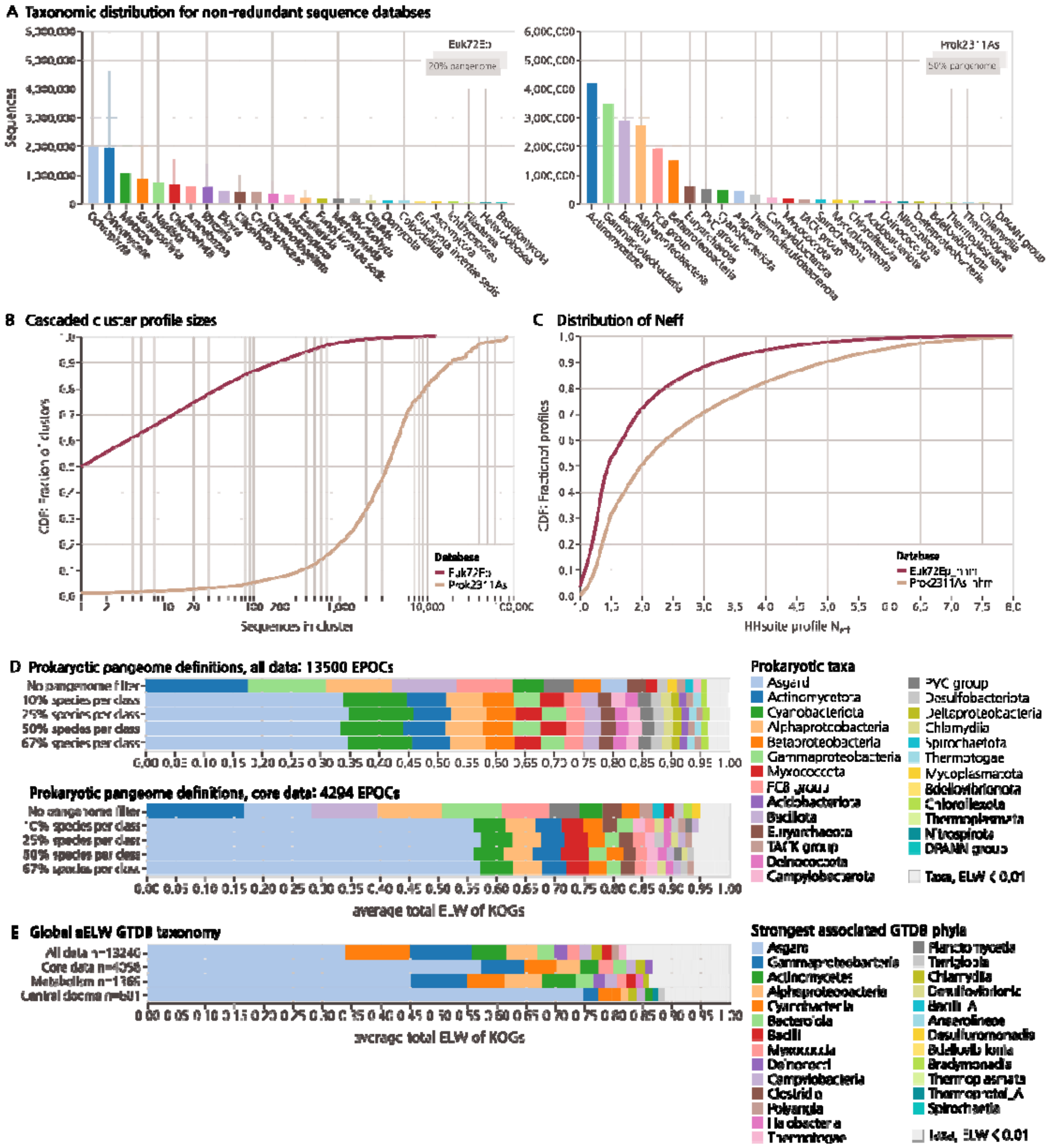

Supplementary Figure 1. Global database statistics. A) Distribution of taxonomic labels present within non-redundant Euk72Ep and Prok2111As (see Methods). Euk72Ep limited to only display taxa with more than 100.000 sequences (99.69% of sequences). **B)** CDF fraction of cluster size distribution following cascaded mmseqs profile/sequence clustering. **C)** CDF fraction of N_{eff} values as calculated by HH-suite for resulting profile databases. **D)** The effect of different pangenome regimes on final aELW values. Pangenomes are reconstructed enforcing a protein presence across 0, 25, 50, or 67% of species within clades and data shown for the full dataset of 14400 EPOCs or core dataset, as described in methods. 50% criteria marked in bold as it is used as reference for the core data present throughout the paper. **E)** Global aELW for data remapped to the GTDB taxonomy at the level of Phylum using marker genes (see Methods). Displaying the top 26 strongest individual contributing phyla out of 92 accounting for 96.4% of all ELW.

bioRxiv preprint doi: https://doi.org/10.1101/2024.10.14.618318; this version posted July 14, 2025. The copyright holder for this preprint (which was not certified by peer review) is the author/funder. This article is a US Government work. It is not subject to copyright under 17 USC 105 and is also made available for use under a CC0 license.

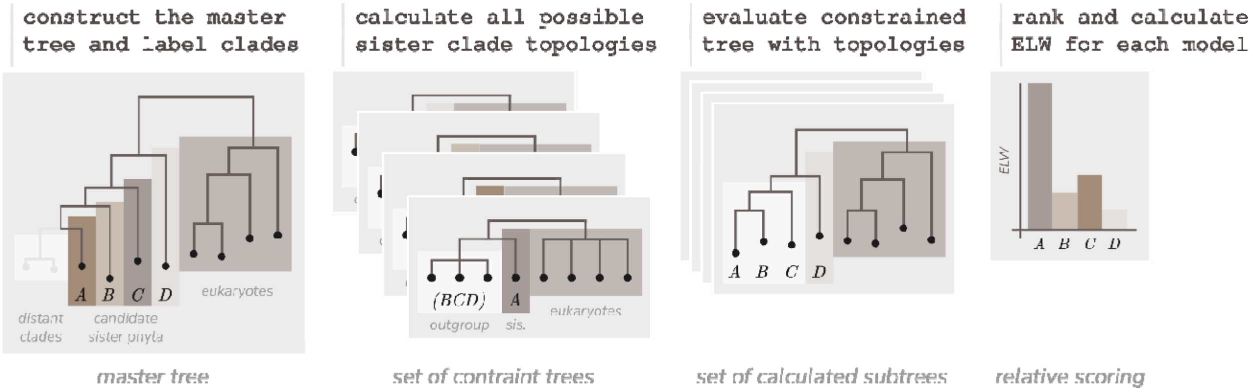

Supplementary Figure 2. Evolutionary hypothesis testing constraint trees and Expected Likelihood Weights. Overview of ELW calculation procedure. One unrooted master tree is constructed from all sequences within a EPOC and the closest n sister clades to the eukaryotic outgroup identified. A set of unrooted constraint trees are generated, each enforcing three clades, one eukaryotic, one sample sister and one outgroup with remaining prokaryotic sequences, is present. The set of subtrees are then constructed using IQtree, each forced to conform to a single guide tree. Log likelihood values for resulting trees are compared and evaluated using IQtree -z to produce confidence sets and Expected Likelihood Weight estimations.

bioRxiv preprint doi: https://doi.org/10.1101/2024.10.14.618318; this version posted July 14, 2025. The copyright holder for this preprint (which was not certified by peer review) is the author/funder. This article is a US Government work. It is not subject to copyright under 17 USC 105 and is also made available for use under a CC0 license.

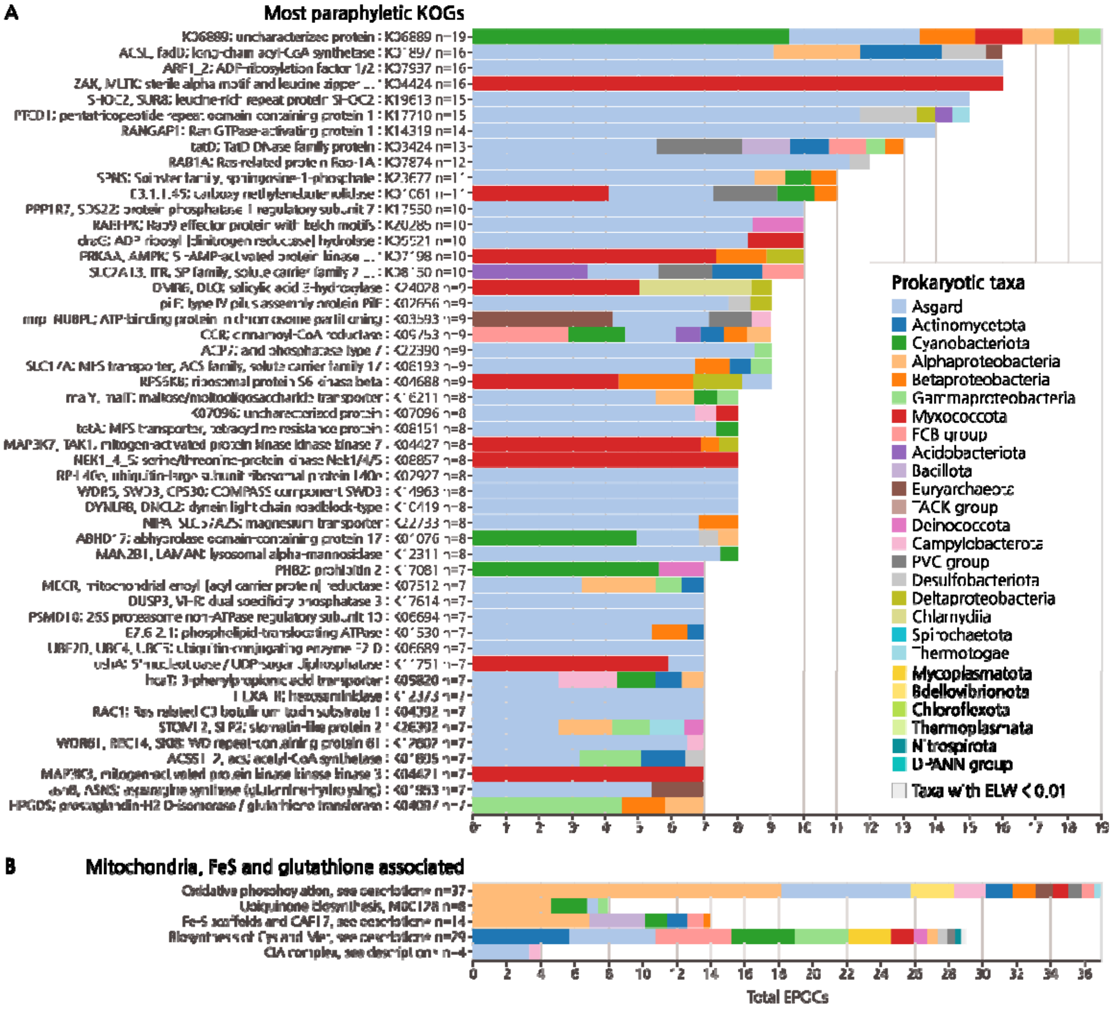

Supplementary Figure 3. aELW breakdown of additional maps and pathways with prominent Asgard and Alphaproteobacterial contributions. A) Overview of most paralogous KOGs estimated by number of separate EPOCs traceable to the LECA. **B)** Oxidative phosphorylation (map00190) also contains entries for V-type ATPases which are excluded here as they are not part of mitochondrial metabolism. Fe-S scaffolds are not members of any map the manually curated set includes KOGs: K22063, K22072, K22068, K22073, K22070, K22071, K04043, K04044 and K04082. The CIA complex is likewise not grouped within BRITE and the manual set contains KOGs: K24730, K26403 and K15075.

bioRxiv preprint doi: https://doi.org/10.1101/2024.10.14.618318; this version posted July 14, 2025. The copyright holder for this preprint (which was not certified by peer review) is the author/funder. This article is a US Government work. It is not subject to copyright under 17 USC 105 and is also made available for use under a CC0 license.

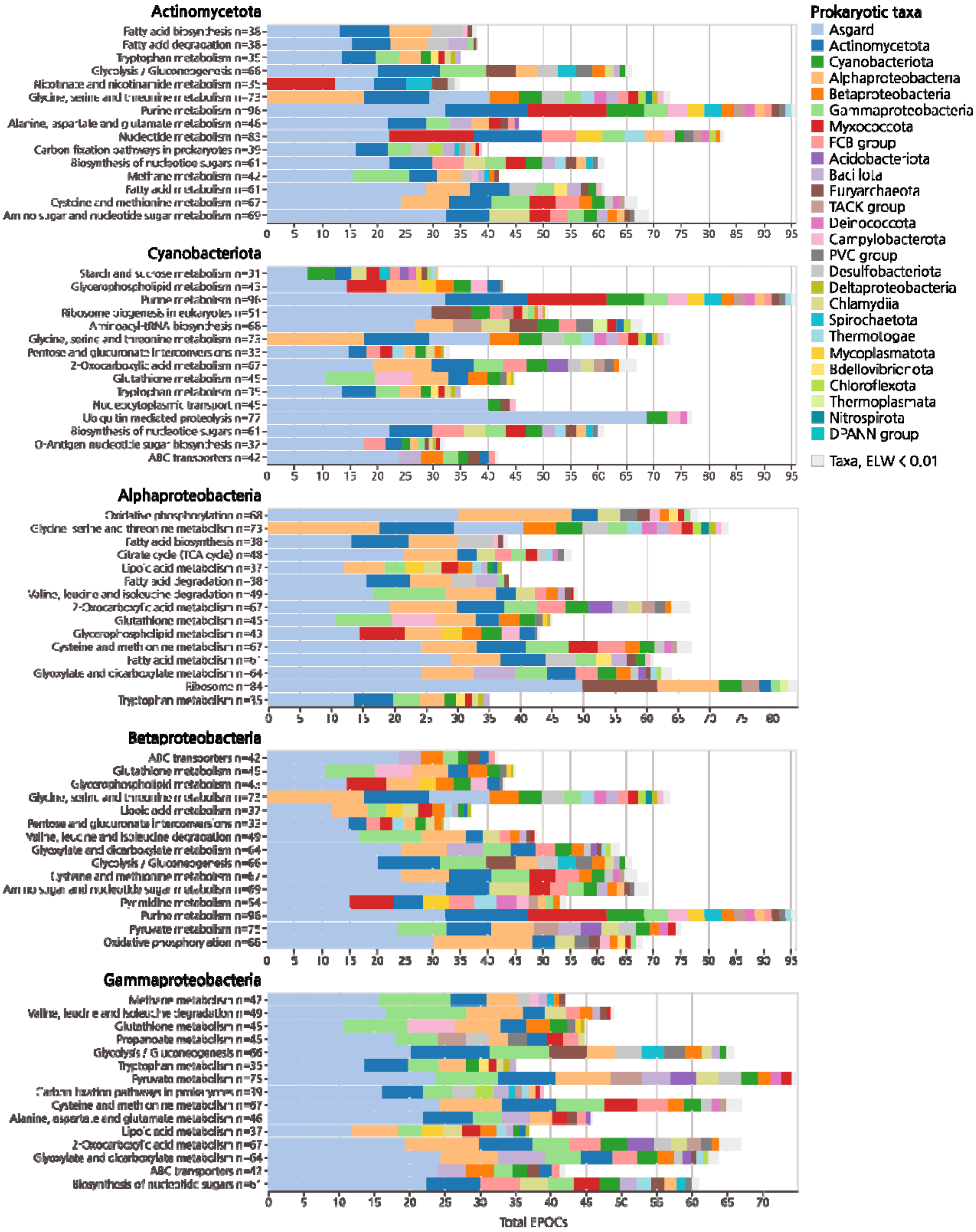

Supplementary Figure 4. Strongest per taxa association of diverse bacteria to eukaryotes. Top bacterial taxa associated taxa with aELW presented for their 15 most prominent pathways in KEGG Brite B. Pathways with more than 20 EPOCs shown.

bioRxiv preprint doi: https://doi.org/10.1101/2024.10.14.618318; this version posted July 14, 2025. The copyright holder for this preprint (which was not certified by peer review) is the author/funder. This article is a US Government work. It is not subject to copyright under 17 USC 105 and is also made available for use under a CC0 license.

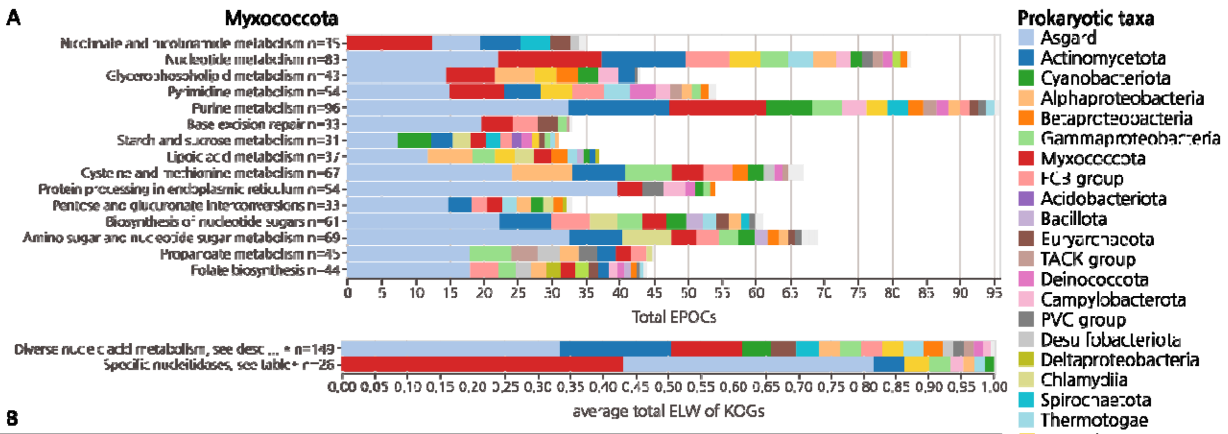

B

KOG	Gene Name	Description	EC number(s)
K10807	RRM1	ribonucleoside-diphosphate reductase subunit M1	1.17.4.1
K01517	ADPRM	manganese-dependent ADP-ribose/CDP-alcohol diphosphatase	3.6.1.13, 3.6.1.16, 3.6.1.53
K01081	E3.1.3.5	5'-nucleotidase	3.1.3.5
K01119	cpdB	2',3'-cyclic-nucleotide 2'-phosphodiesterase / 3'-nucleotidase	3.1.4.16, 3.1.3.6
K19970	NT5E, CD73	5'-nucleotidase	3.1.3.5
K03742	pncC	nicotinamide-nucleotide amidase	3.5.1.42
K03462	NAMPT	nicotinamide phosphoribosyltransferase	2.4.2.12
K11751	ushA	5'-nucleotidase / UDP-sugar diphosphatase	3.1.3.5, 3.6.1.45
K01525	apaH	bis(5'-nucleosyl)-tetraphosphatase (symmetrical)	3.6.1.41
K03651	cpdA	3',5'-cyclic-AMP phosphodiesterase	3.1.4.53
**K12410**	**cobB, srtN, npdA**	**NAD-dependent protein deacetylase/lipoamidase**	**2.3.1.286, 2.3.1.313**

Supplementary Figure 5. Sparse Myxococcota association with eukaryotes. A) Top, aELW breakdown of enriched Myxococcota pathways showing primary association with nucleotide synthesis. Bottom, curated set of Myxococcota enriched EPOCs containing general nucleic acid metabolism and modification pathways corresponding to KEGG map00760, map01232, map00240 and map00230 with notable presence of Myxococcota. Further selection of only those KOGs within the above pathways which include Myxococcota. **B)** List of KOGs with Myxococcota presence within the curated set of nucleotide synthesis highlighting phosphatases and phosphotransferases.

bioRxiv preprint doi: https://doi.org/10.1101/2024.10.14.618318; this version posted July 14, 2025. The copyright holder for this preprint (which was not certified by peer review) is the author/funder. This article is a US Government work. It is not subject to copyright under 17 USC 105 and is also made available for use under a CC0 license.

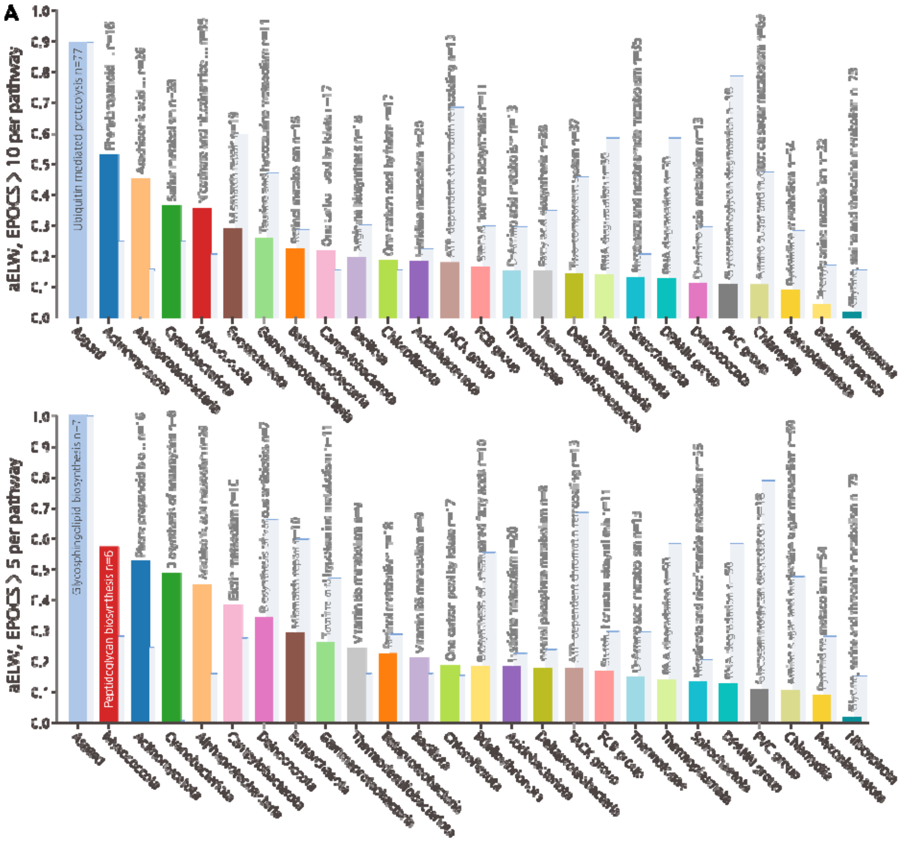

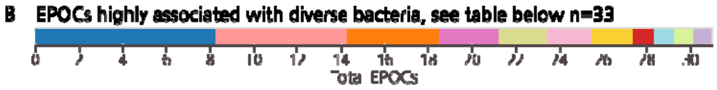

KOG	Gene Name	Description	EC number(s)
K02564	nagB, GNPDA	glucosamine-6-phosphate deaminase	3.5.99.6
K12862	PLRG1, PRL1, PRP46	pleiotropic regulator 1	
K07198	PRKAA, AMPK	5'-AMP-activated protein kinase, catalytic alpha subunit	2.7.11.31
K10570	ERCC8, CKN1, CSA	DNA excision repair protein ERCC-8	
K06972	PITRM1, PreP, CYM1	presequence protease	3.4.24.-
K05967	K05967	uncharacterized protein	
K03781	katE, CAT, cat3, srpA	catalase	1.11.1.6
K04688	RPS6KB	ribosomal protein S6 kinase beta	2.7.11.1
K01908	ACSS3, prpE	propionyl-CoA synthetase	6.2.1.17
K13137	STRAP, UNRIP	serine-threonine kinase receptor-associated protein	
K06013	STE24	STE24 endopeptidase	3.4.24.84
K14085	ALDH7A1	aldehyde dehydrogenase family 7 member A1	1.2.1.31, 1.2.1.8, 1.2.1.3
K00597	MTRR	methionine synthase reductase	1.16.1.8
K00023	phbB	acetoacetyl-CoA reductase	1.1.1.36
K14430	PHO87_91	phosphate transporter	
K08956	AFG3	AFG3 family protein	3.4.24.-
K00261	GLUD1_2, gdhA	glutamate dehydrogenase (NAD(P)+)	1.4.1.3
K12450	RHM	UDP-glucose 4,6-dehydratase	4.2.1.76
K00281	GLDC, gcvP	glycine cleavage system P protein (glycine dehydrogenase)	1.4.4.2
K11141	ENPEP, CD249	glutamyl aminopeptidase	3.4.11.7

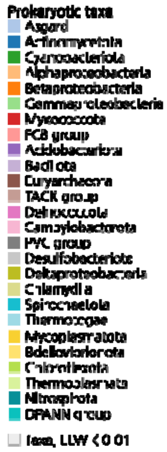

Supplementary Figure 6. KOGs of strong bacterial association. A) Same as main figure 4 under less stringent selection criteria indicating most prominent prokaryotic pathways with a coverage of at least 10 and 5 EPOCs, aELW shown relative to that of Asgard for the same pathway in pale blue. **B)** Individual EPOCs strongly associated with diverse prokaryotic taxa under stringent selection criteria. EPOCs must contain a eukaryotic outgroup with at least 15 unique taxa encompassing the LECA, have a prokaryotic sister clade with an ELW of > 0.7 which is not derived from Asgard, Euryarchaeota, the TACK group, DPANN group, Cyanobacteriota or Alphaproteobacteria and contain more than 20 sequences. The EPOCs which meet these criteria are highlighted with respective KOGs indicated, aELW displayed.

bioRxiv preprint doi: https://doi.org/10.1101/2024.10.14.618318; this version posted July 14, 2025. The copyright holder for this preprint (which was not certified by peer review) is the author/funder. This article is a US Government work. It is not subject to copyright under 17 USC 105 and is also made available for use under a CC0 license.

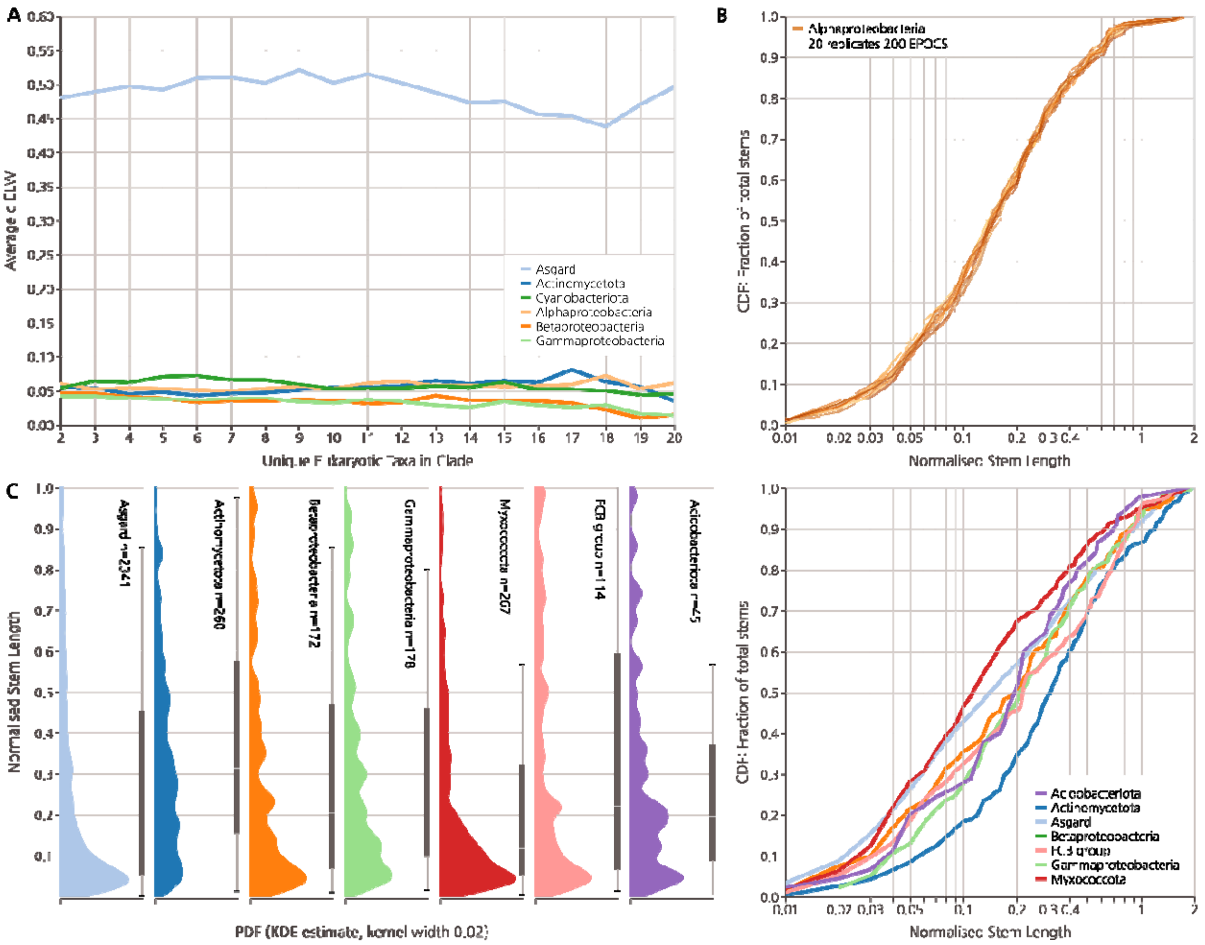

Supplementary Figure 7: **Clade scope definition and stem length distribution for diverse bacteria compared to Asgard**. **A)** Global aELW dependence on choice of clade size cutoff using core data. Estimated aELW are independent on choice of eukaryotic clade scope. **B)** 20 full technical replicates, including muscle5 alignment, IQtree2 calculations and constraint tree generation, of 175 randomly sampled Alphaproteobacterial stems showing reproducibility of stem length distributions. Compared with distributions drawn from Asgard and Cyanobacterial stems as per Figure 5. **C)** Left, Probability Density Function (PDF) of normalized stem lengths taken from samples of individual core eukaryotic genes associated with prokaryotic taxa, obtained through KDE. Thin bar indicates 5th percentile ranges, thick bar includes 25th percentile ranges with median indicated. Right, fractional cumulative distribution functions of normalized stem lengths.

bioRxiv preprint doi: https://doi.org/10.1101/2024.10.14.618318; this version posted July 14, 2025. The copyright holder for this preprint (which was not certified by peer review) is the author/funder. This article is a US Government work. It is not subject to copyright under 17 USC 105 and is also made available for use under a CC0 license.

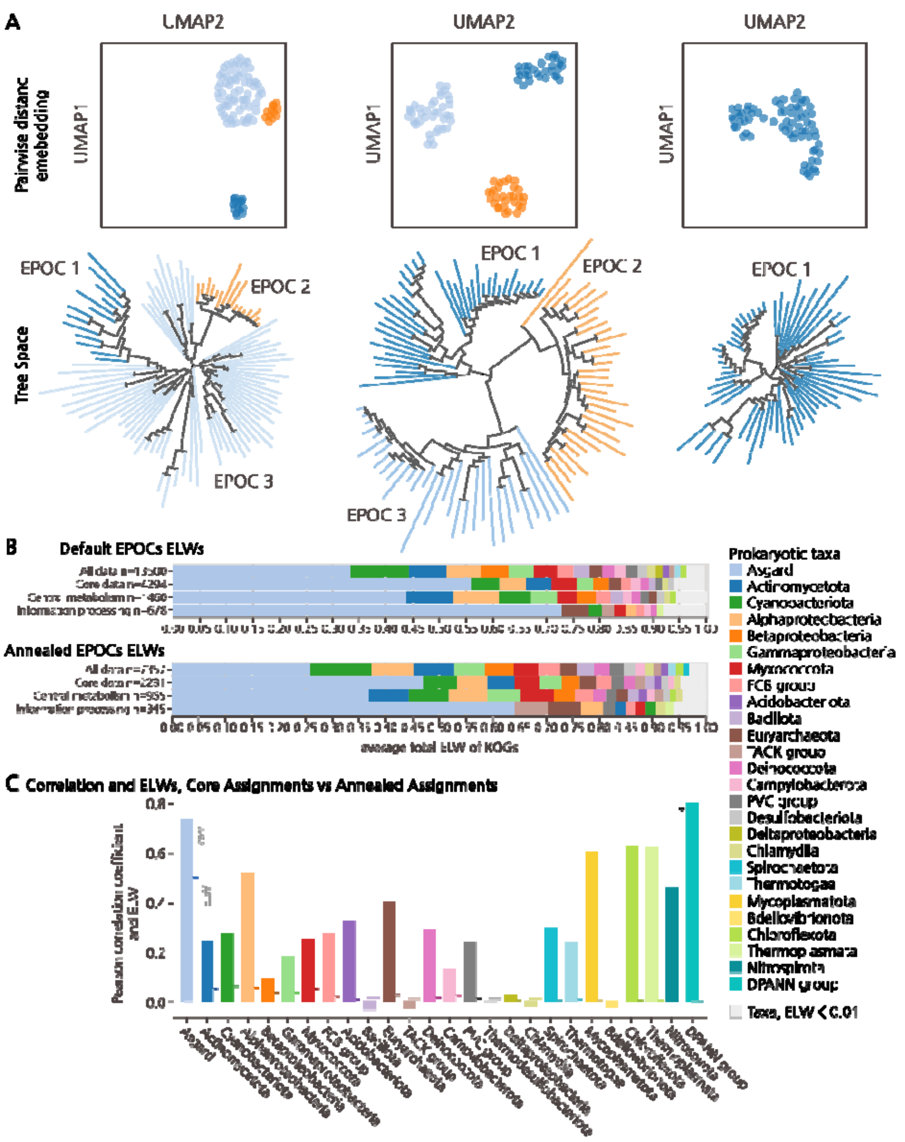

Supplementary Figure 8: **Annealed cluster EPOCS A)** Examples of annealed clades defined based on embedded and clustered pairwise distance matrices of phylogenetic trees. Top, embedding of pairwise leaf distance of phylogenetic tree, bottom. Clustered by HDBSCAN allowing single cluster partitions as shown, left. Partitions form monophyletic subsets of well partitioned branched. **B)** Resulting top level aELW values derived from the core data clusters as well as annealed EPOCs showing broad correlation between different core and annealed partitioning schemes. **C)** Correlation of aELW vectors calculated for all Prokaryotic taxa across all KEGG maps using the annealed and core EPOCs indicating good agreement for Asgard assignments but greater variance between prokaryotic taxa. Overlaid on global aELW values. Taxa for which aELW is less than 0.01 are found to be associated only for very few pathways and as such correlate strongly, notably DPANN has an aELW of 0 and do not contribute to any pathways, and therefore with a correlation of 1.0 between the two reconstructions.
